# Synthesis of a new hydrophobic coating film from stearic acid of buffalo fat

**DOI:** 10.1038/s41598-022-23003-4

**Published:** 2022-11-02

**Authors:** Hanaa M. Soliman, Hamdy A. Zahran

**Affiliations:** Fats and Oils Department, Food Industries and Nutrition Research Institute, National Research Centre, Dokki, Cairo, 12622 Egypt

**Keywords:** Lipids, Biotechnology

## Abstract

This experiment involved the chemical conversion of pure stearic acid from buffalo adipose tissue to a waxy stearyl stearate, which was subsequently applied as a coating film to extend the shelf life of recently harvested fruits. Fat was extracted from minced adipose tissue using the dry rendering procedure, and it was then characterized. The extracted fat was hydrolyzed into a mixture of free fatty acids and glycerol. The supercritical CO_2_ extractor was used for stearic acid individual extraction in pure form from the free fatty acid mixture, and it was confirmed according to its melting point (69.2–70.0 °C), elemental analysis, GC–MS for esterified fatty acids. The isolated stearic acid was used for the synthesis of a new hydrophobic wax named stearyl stearate. The chemical structure of the prepared compound was established according to its elemental analysis and spectral data. The new hydrophobic wax was used as a coating film to enhance the shelf life of freshly harvested tomato fruits. Therefore, stearyl stearate solution (2.00% w/v diethyl ether) was used for tomato coating and compared to chitosan-coated tomatoes, where weight loss, pH, fruit firmness, ascorbic acid concentration, and total soluble solids were studied for a period of 15 days at 23 ± 1.0 °C and 65 ± 2.0% relative humidity. The results revealed that coating with stearyl stearate solution (2.00% w/v diethyl ether) could delay tomatoes’ ripening during the experiment condition. A sensory evaluation of the coated tomatoes was carried out and showed acceptable taste for the tomatoes that were coated with stearyl stearate. On the other hand, the acute oral toxicity of stearyl stearate using albino mice showed complete safety up to 25 g/kg mice weight.

## Introduction

Fruits and vegetables are known for their high nutritional benefits as they contain many valuable chemical compounds such as citric acid, ascorbic and other organic acids, sugars, minerals, vitamins, carotenoids, and phenolic compounds^1^. Some of these compounds have physiological properties, including anti-inflammatory, anti-allergic, antimicrobial, vasodilating, anticoagulant, heart disease prevention, and antioxidant^2^. Tomatoes are one of the most widely used fruits because of their distinctive taste and many benefits.

Therefore, consumers are accustomed to using tomatoes in processed products such as soups, juices, sauces, and for cooking because of its delicious flavor, and in its fresh form. Moreover, it is widely used in the food industry as a raw material for the production of derivative products^3^. Fruits and vegetables are extremely sensitive products to shocks, vibration, temperature, and humidity during transportation, storage, and marketing processes^4^. On the other hand, due to their relatively short lives, their transportation over long distances is limited, and they cannot be available around the year^5^.

Post-harvest fruit losses are a serious problem mainly caused by physiological disturbances and a lack of proper food storage techniques resulting in significant economic losses. Packaging materials protect and preserve food in order to improve its quality and shelf life, facilitating its marketing and distribution^6^. The food industry has been forced to develop new and better ways to extend shelf life and maintain food quality due to the growing demand for fresh fruits and vegetables^7^. Recently, loss of firmness, moisture, solute migration, gas exchange, and physiological disorders have been prevented by controlling respiration rate and maturity through the developed coating films and coatings, which act as a permeable barrier against moisture and some other solute movement^8^.

Due to their eco-friendliness and minimal reliance on non-renewable resources, biodegradable films and coatings are viable alternatives to synthetic films in the packaging sector and have caught the interest of numerous academics^9,10^. Food goods are shielded from mechanical, physical, and chemical harm and quality loss by biodegradable packaging. Additionally, it extends the shelf life of food goods and acts as an antimicrobial barrier in the form of antimicrobial packaging to inhibit microbiological activity^11,12^. Based on their ingredients and place of origin, edible coatings are classified into natural or synthetic. Some synthetic films are manufactured from petrochemical basic materials, while others are natural and derived from oligosaccharides (starch and cellulose), proteins like gelatin, and milk protein^13,14^.

The purpose of this study was to prepare a new hydrophobic coating film with a waxy texture, in order to be able for fruit coating, and to control the water permeability through the fruit epidermis, thus enhancing the shelf life of the freshly harvested fruits. And it was achieved via preparation of stearyl stearate, due to absence of any unsaturation center with in the very long hydrocarbon chain. In addition, buffalo fat can be considered as a byproduct, and it well known by its high concentration of stearic acid. Thus, buffalo fat was used as a cheap source for our synthesis. So, freshly harvested tomatoes were chosen as a typical example for enhancing its shelf life using the prepared film.

## Experiment

### Instruments

Melting points were measured on an Electrothermal™ IA9300 Beginning Ending Recording Model for Pharmacopeia Requirements. Capillary gas chromatograph (HP 6890) was used for the qualitative and quantitative determinations of fatty acids of the extracted fat samples and reported in relative area percentages. Supercritical CO_2_ instrument used in the present study was applied, Separations. Inc., Allentown, USA, model no.7071. The NMR spectra were recorded on a Varian Mercury VX-300 NMR spectrometer. ^1^H spectra were ran at 300 MHz. Chemical shifts are quoted in δ and were related to that of the solvents. Mass spectra were recorded on a Shimadzu GC MS-QP 1000 EX mass spectrometer at 70 eV. Elemental analyses were carried out at the Micro-analytical Center of Cairo University. Portable hyper-spectrometer (Spectronic 21D, milton roy boulder, Colorado, USA). PH meter (Pinnacle 530 Bench top pH meter). Fruit firmness was determined using Universal Testing Machine (Zwick GmbH & Co, Germany).

### Material and reagents

Buffalo adipose tissue was obtained from local market and chilled in ice while transporting to the laboratory. It was washed, dried with a towel and weighed for further use. Fresh tomato fruits were obtained from local market. All solvents were HPLC grade and were supplied from Merck (Darmstadt, Germany), and having a purity of ~ 99%.

### Fat extraction from buffalo adipose tissue

Buffalo adipose tissue (500 g) was minced, lipids were extracted from fatty tissues by dry rendering method^15^ at 90 °C for 3 h. After cooling to 50 °C, fat was filtered off through Whatman No. 1 filter paper. Then it was kept in a brown bottle at 5 °C until analysis.

### Quality parameters

#### Refractive index (RI)

The refractive indexes (RI) of oil samples were determined using a refractometer (Rudolph model J157 at 20 °C, each test was performed five repetitions. It was determined according to the method described by AOAC^16^.

#### Acid value (AV)

The method used was adapted from AOAC^16^. A mixture of absolute ethanol and diethyl ether (1:1 v/v) was carefully neutralized with 0.10 N potassium hydroxide solution using 1% phenolphthalein indicator. About 5 g of the tested samples were dissolved in 50 ml neutralized ethanol-diethyl ether solvent and titrated with 0.10 M potassium hydroxide with constant shaking until a pink color persisted for 15 s. Free fatty acids were calculated as % of oleic acid.

$$FFA \, \% \, as \, oleic \, acid \, = \, S \, \times \, 0.0282 \, \times \, 100/W \, \left[ \% \right],$$

where *S* titration (ml), *W* weight of the oil (g).

#### Iodine value (IV)

The Iodine value of the tested samples was determined using Hanus method according to the method described in AOAC^16^. Briefly, about 0.25 g of tested sample was weighted into 500 ml glass stopper flask and dissolved in 20 ml chloroform. 25 ml Hanus iodine (13.2 g pure I_2_ in 1 l CH_3_COOH) solution was added and let stand 30 min in dark. 10 ml 15% KI solution was added shacked thoroughly and then100 ml freshly boiled and cooled H2O was added. I_2_ was titrated with 0.10 N sodium thiosulphate solution with constant shaking until yellow solution turns almost colorless. A few drops of 1% starch solution as indicator was added and titration was continued until blue entirely disappears with shaking vigorously to release all iodine from CHCl_3_. A blank was performed omitting the oil, and the iodine value was calculated as grams of iodine per 100 g of oil.

$$Iodine \, value \, = \, \left( {B \, {-} \, S} \right) \times N \times 12.69/W \, \left[ {{\text{g}}/100\;{\text{g}}} \right],$$

where *B* mL Na_2_S_2_O_3_ solution required for blank, *S* mL Na_2_S_2_O_3_ solution required for test sample, *N* normality of Na_2_S_2_O_3_ solution, and *W* weight of sample in g.

#### Peroxide value (PV)

The PV was determined according to the AOAC^16^. Tow g from tested oil were weighted in flask with ground-glass cap. 250–300 ml, 10 ml of chloroform were added and shacked for 10 min, 15 ml of glacial acetic acid, and 2 g of sodium bicarbonate NaHCO_3_ were added, after stirring, 1 ml of a saturated solution of KI was added and shacked for 1 min and kept in dark place for 5 min, immediately. After the specified time, 75 ml distilled water, then 0.5 ml of starch solution were added, the resulting solution was titrated with a solution of Na_2_S_2_O_3_ (0.01 N) to the disappearance of the blue color, the blank was carried on the same procedures but without sample. A peroxide value of the tested oil given by the equation:

$$PV = \, [\left( {V1 - V2} \right) \times N \times 1000] / \, W \left[ {{\text{meq}}{\text{.O}}_{2} /{\text{kg}}} \right],$$

where *V1* volume of sodium thiosulfate solution consumed in the titration of sample (ml), *V2* volume of sodium thiosulfate consumed in the titration of the blank (ml), *N* the normality of sodium thiosulfate, *W* weight of fat taken to denote (g).

#### Determination of unsaponifiable matter content

Saponification value of the tested oil samples was determined according to the method described in AOAC^16^. Briefly, 5 g of the tested sample was weighted into 250–300 ml conical flask, and 50 ml alcoholic KOH solution (35–40 g KOH were dissolved in 20 ml water and diluted to one liter with alcohol, 95%) was added. Flask was connected with air condenser, boiled until fat was completely saponified (~ 30 min), cooled and titrated with 0.5 M HCl using phenolphthalein. Saponification value was calculated by the following formula:

$$Saponification \, value \, = \, 28.05 \, \left( {B{-}S} \right){/}W\left[ {{\text{mg}}/{\text{g}}} \right],$$

where *B* mL HCl required for blank, *S* mL HCl required for test sample, *W* weight of the sample in g.

### Fatty acids composition

The fatty acids composition of the extracted fat was determined by GC–MS after their esterification^17^. The extracted fat was esterified into the corresponding methyl esters by shaking a solution of fat (0.1 g) in heptane (2 mL) with methanolic potassium hydroxide solution (0.2 mL, 2 N). The produced fatty acid methyl esters were identified using gas chromatograph. Nitrogen flow rate was 0.6 ml/min, hydrogen and air-flow rates were 45 and 450 mL/min, respectively. The oven temperature was isothermally heated to 195 °C. The injector and the detector temperatures were 230 °C and 250 °C, respectively. Fatty acid methyl esters were identified by comparing their retention times with known fatty acid standard mixture. Peak areas were automatically computed by an integrator.

### Oil hydrolysis

It’s well-known that, fat molecule is a triacyl glycerol, so it could be easily hydrolyzed in an aquatic medium at high temperature and pressure to produce fatty acids mixture and glycerol^18–20^. Thus, the extracted fat (418.75 g) was hydrolyzed with distilled water (1.5 L) in a high-pressure reactor at 250 °C and 2 MPa_,_ the reaction was left to cool to room temperature, where the reaction mixture was separated into two layers. The upper layer contained the fatty acids mixture, while the lower one contained water, glycerol and traces of non-fatty materials. The upper layer was separated, dried over anhydrous sodium sulphate, filtered off and weighted.

### Confirmation of oil hydrolysis

#### Thin layer chromatographic analysis (TLC)

Oil hydrolysis was confirmed by thin layer chromatographic analysis (TLC), where, plates (20 × 20) were coated with a slurry of silica gel (60 G) in water (15 g/hg), left to dry, then it was activated at 110 °C for 1.0 h. Standard spot of known fatty acid and other spot of oil were spotted (individually) on the activated thin layer plates, at the base line (2 cm from the bottom). The fatty acids mixture of the hydrolyzed fat was also spotted on the same line. The developing solvent consisted of n-hexane, diethyl ether and acetic acid at a volumetric ratio 80:20:1, respectively. The developing jar was lined on three sides with filter paper wetted with the same developing solvent. The plates were developed till the solvent reached the front line (15 cm from the start line). The spots of different components separated by TLC were then visualized by iodine vapor. The fatty acids were considered formed when the withdrawn sample showed only one spot with no tail, and with rate of flow similar to that of the known fatty acid spot but not the oil spot.

#### ^1^H NMR spectrum

The fatty acids mixture of the hydrolyzed fat was also confirmed according to ^1^H NMR spectrum, where signal at δ11.01 was observed which indicates the presence of carboxylic hydrogen of the free fatty acids.

#### Extraction of stearic acids using supercritical CO_2_ extractor

Pure stearic acid was extracted individually from the free fatty acids mixture of buffalo adipose tissue using supercritical CO_2_ extractor^21^ at 40.0 MPa and 328 K, and it was confirmed depending on its chemical analysis (C, 76.00; H, 12.76%), milting point (69.2–70 °C) and GC/MS of its corresponding ester.

Yield (125.17 g, 98%), m.p. (69.2–70 °C), IR (KBr) ν_max_/cm^−1^: 2792(OH), 2981–2840 (CH-aliphatic), 1773 (C=O). ^1^H NMR (CDCl3): δ 11.1(s, 1H), 2.19(t, 2H), 1.55(m, 2H), 1.28–1.32(m, 28H), 0.96(t, 3H). 13C NMR (DMSO—d_6_): δ14.1, 22.7, 24.8, 29.1, 29.4, 29.7, 31.2, 36.0, 177.5. For C_18_H_36_O_2_ (284.48): Calcd.: C, 76.00; H, 12.76%. Found: C, 76.07; H, 12.70%.

#### Synthesis of a new hydrophobic wax (stearyl stearate)

Stearyl stearate is an ester of wax like properties. Thus, it could be prepared via interaction between stearic acid and stearyl alcohol in a catalytic medium. So, a portion of the extracted stearic acid underwent reduction process in order to afford the corresponding alcohol.

#### Preparation of stearyl alcohol

Stearic acid (56.9 g, 0.2 mol) was reduced to stearyl alcohol^22^ under mild conditions of temperature (140–180 °C) and H_2_ pressure (5 MPa) using water as a solvent, over N-modified carbon (N–C) supported with Ru and Sn catalysts (RuSn/N–C). The N–C support was prepared using one-pot process with melamine, glucose, and ZnCl_2_ at vigorous temperature (800 °C) and N_2_ gas for 1 h in presence of Ru and Sn. The un-reacted acid and the produced alcohol were separated from each other using separating funnel, where Na_2_CO_3_ solution (25 mL, 2 N) was added in order to convert acid into water soluble salt (soap), thus water insoluble stearyl alcohol could be separated, dried over anhydrous sodium sulphate, filtered off, and confirmed according to its milting point, chemical analysis and spectral data.

Yield (44. 36 g, 82%), m.p. (59.4–60 °C), IR (KBr) ν_max_/cm^–1^: 3604(OH), 2920–2842 (CH- aliphatic), 1035 (C–O). ^1^H NMR (CDCl3): δ 3.51(t, 2H), 2.1(s, 1H), 1.45(m, 2H), 1.28–1.32(m, 30H), 0.95(t, 3H). 13C NMR (DMSO—d_6_): δ14.1, 25.8, 29.4, 29.9, 31.9, 32.4, 62.8. For C_18_H_38_O (270.49): Calcd: C, 79.93; H, 14.16%. Found: C, 79.88; H, 14.19%.

#### Preparation of stearyl stearate

Production of stearyl stearate from stearic acid (42.07 g, 0.148 mol) and stearyl alcohol (40.00 g, 0.148 mol) was performed in diethyl ether (200 ml), and in the presence of KOH as a base catalyst at 3% of the mixture weight. The reaction mixture was refluxed for 6 h. This mixture was then washed with warm distilled water, where two layers were formed, so the lower one was removed in order to get rid of the water-soluble catalyst, the warm fatty layer was dried over anhydrous sodium sulfate, filtered off and collected. Then stearyl stearate was separated from the fatty mixture according to its melting point.

#### Separation of stearyl stearate from the fatty mixture

The pervious formed fatty mixture contained deferent fatty compounds with deferent melting points (ester, unreacted fatty acid and unreacted fatty alcohol). The fatty mixture was cooled in a refrigerator for 72 h, then the temperature was gradually raised to 60.3 °C, so only stearyl alcohol would melt and thus be removed by filtration. Increasing the temperature to 63 °C caused the stearyl ester to melt, so that it could be collected and separated from the stearic acid by filtration. The separated ester was confirmed according to its chemical analysis, spectral data and milting point.

Yield (69.03 g, 87%), m.p. (62–63 °C), Shape, yellowish white waxy platelets, IR (KBr) ν_max_/cm^−1^: 2920–2842 (CH-aliphatic), 1737 (C=O), 1154(C–O). ^1^H NMR (CDCl3): δ 4.06(t, 2H), 2.26(t, 2H), 1.66(m, 2H), 1.29–1.33(m, 58H), 0.96(t, 6H). 13C NMR (DMSO—d_6_): δ14.1, 22.6, 25.1, 25.3, 29.1, 29.2, 29.4, 29.5, 29.7, 29.9, 31.9, 65.4, 133.9, 173.2. For C_18_H_38_O (270.49): Calcd: C, 80.53; H, 13.52%. Found: C, 80.38; H, 13.79%.

### Coating application

Sixty post-harvested tomatoes fruits (selected based on uniformity of size and ripening stage with absence of physical damage and fungal infection) were washed with chlorinated water (200 ppm) for 3 min, left to dry at room temperature for 1 h, then tomatoes were randomly distributed into three groups. Coatings were applied by double immersion of fruits in the film-forming solutions for 5 min. The depending solutions were: (i) stearyl stearate 2.00% (w/v diethyl ether), (ii) chitosan 2.00% (w/v lactic acid) and (iii) control sample (treated with water). Fruits were allowed to dry at room temperature for 1 h. The experiment was conducted at ambient condition (20–25 °C with 65–85% relative humidity) for 15 days.

### Quality attributes

#### Weight loss

Change in tomatoes weight during the storage period was calculated^22^ by weighing the fruit on a digital balance with specific time interval (3 days). The results were represented as percent weight loss.

#### pH

The pH of the blended samples was determined by a standard calibrated digital pH meter (Pinnacle 530 Bench top pH meter).

#### Fruit firmness

Fruit firmness was determined^22^ by compression test using Universal Testing Machine (Zwick GmbH & Co, Germany). Tomatoes were compressed by a cylindrical probe of 10 mm diameter at the test speed of 10 mm/min. The firmness of the fruit was taken as the slope of linear section of force deformation curve (N/mm).

#### Ascorbic acid concentration

The ascorbic acid level in the tomato fruits was determined^23^. In brief, 10 g of tomato fruit pulp was homogenized with 100 ml of distilled water. Then, 10 mL of this homogenate was mixed with 10 mL of 20% meta-phosphoric acid and the final volume was adjusted to 100 mL with distilled water. The 2,6-dichlorophenol indophenol dye was used for titration of the solution. The following formula was adopted for calculation of the ascorbic acid level in each sample: Ascorbic acid (mg/100 ml homogenate; i.e. mg/10 g fruit pulp) = (Titre × dye factor volume made up × 100)/(volume taken for titration × sample weight). The ascorbic acid content was expressed as milligrams per 10 g (mg/10 g) of fruit pulp.

#### Total soluble solid (TSS)

Soluble solids are free sugar molecules resulting from enzymatic hydrolysis of polysaccharides. And could be determined as °Brix, using the refractive index measurements. Total soluble solid (TSS) of tomato juice was measured by using a digital refractometer (RX-5000i-plus, Atago, Japan) at 20 °C, and the results were expressed as °Brix^24^.

#### Sensory evaluation

The sensory properties of uncoated and coated (stearyl stearate and chitosan) tomatoes were evaluated subjectively by using a 9-point hedonic scale. The evaluation includes the appearance, flavor, texture and overall acceptability of coated and uncoated tomatoes^25^.

##### Acute toxicity test

Albino mice of 22–25 g weight were housed individually under standard conditions (12-h light/dark cycle; 25 ± 3 °C temperature; 35–60 relative humidity). Thirty animals were divided into six groups (5 animals/group); one group was used as control and fed on standard mice feed only, and the other was given a mixture of oral doses of stearyl stearate and standard mice feed^26^. The experiment was performed following the National Research Centre’s Medical Research Ethics Committee (MREC) ethical guidelines for regular experimental animal studies, and the committee approved the experimental protocol, which complied with the instructions provided by the Guide for Care and Use of Laboratory Animals (U.S.—N.I.H. Publication No. 85-23, revised 1996) and the ARRIVE guidelines (Animal Research: Reporting of In Vivo Experiments). The experiment was also carried out under the U.K. Animals (Scientific Procedures) Act 1986 (This Act regulates the use of protected animals in any experimental or other scientific procedure which may cause pain, suffering, distress or lasting harm to the animal. Protected animals under the Act are any living vertebrae other than man and any living cephalopod) and associated guidelines, Directive 2010/63/E.U., revising Directive 86/609/E.E.C. on the protection of animals used for scientific purposes was adopted on 22 September 2010. The Directive is firmly based on the principle of the Three Rs, to replace, reduce and refine the use of animals for scientific purposes.

##### Liver and kidney function tests

Liver and kidney function of those Albino mice were tested in order to improve the safety of stearyl stearate as a food additive. Where, Alanine transaminase (ALT), Aspartate transaminase (AST), alkaline phosphates (ALP), Creatinine and Urea were determined.

### Statistical analysis

All experiments and analytical determinations were performed three times. The averages and standard deviation were calculated by statistical analysis using SPSS program 10.0 (IBM Corporation, Armonk NY).

## Results and discussion

### Fat extraction from buffalo adipose tissue

Buffalo Fat (a rich source with stearic acid) was extracted from minced buffalo adipose tissue (500 g) by dry rendering method^15^, and afforded 418.65 g which corresponding to 83.73% as shown in Table 1.

**Table 1:** Fat content (%) and some of its physicochemical properties.

Parameter	Buffalo fat
Fat content (%)	83.73 ± 0.11
Refractive index (at 25 °C)	1.4356 ± 0.09
Free fatty acids (%)	0.34 ± 0.22
Peroxide value (meq/kg oil)	2.9 ± 0.16
Iodine value (g/100 g)	45.7 ± 0.23
Saponification number (mg/g)	203 ± 0.17
Unsaponifiable matter (%)	0.44 ± 0.30

Data are expressed as mean ± SD values given represent means of three determinations.

### Quality parameters

The extracted buffalo fat was characterized physically and chemically^9^, and the results are listed in Table 1. The refractive index, free fatty acids, peroxide value, iodine number, saponification number and unsaponifiable matter was 1.4356, 0.34, 2.90, 45.70, 203 and 0.44% respectively for buffalo fat extract.

### Fatty acids composition

Lipid extracted from buffalo adipose tissue can be used as an available source of fatty acids, which may then be used in various chemical and pharmaceutical reactions, where it was found to contain 13 fatty acids according to GC–MS scan. The predominant fatty acids were stearic acid (C_18:0_) and palmitic acid (C_16:0_), those represent 32.92 and 20.95% respectively. While the dominant unsaturated fatty acids were oleic acid (C_18:1_) and myristoleic acid (C_14:1_) (10.62 and 8.88%) respectively; as illustrated in Table 2. All samples’ significant levels of fatty acids were generally consistent with those from prior research^27^.

**Table 2:** Fatty acid composition of buffalo fat.

Fatty acid	Structure	Relative amount (%)
Lauric acid	C12:0	0.54 ± 0.04
Myristic acid	C14:0	9.92 ± 0.08
Myristoleic	C14:1	8.88 ± 0.12
Palmitic acid	C16:0	20.95 ± 0.45
Palmitoleic acid	C16:1	5.34 ± 0.13
Stearic acid	C18:0	32.92 ± 0.08
Oleic acid	C18:1	10.62 ± 0.47
Linoleic acid	C18:2	4.75 ± 0.43
Linolenic acid	C18:3	4.86 ± 0.02
Arachidic acid	C20:0	0.20 ± 0.10
Behenic acid	C22:0	0.11 ± 0.07
Arachidonic acid	C20:4	0.29 ± 0.08
Paullinic acid	C22:1	0.21 ± 0.00
Others	–	0.41
Saturated fatty acid (%)	–	64.53
Unsaturated fatty acid (%)	–	35.06

Data are expressed as mean ± SD values given represent means of three determinations.

### Oil hydrolysis

Lipid extracted from buffalo adipose tissue (418.68 g, 83.73%) was hydrolyzed^11,13^, where ester bonds of oil molecule were broken by action of temperature and pressure to afford a mixture of free fatty acids (388 g) and glycerol. Fatty acids formation was established according to TLC analysis, where the formed fatty acids mixture showed a unique spot with no tail and with rate of flow (Rf value) similar to that of known fatty acid but not the oil, moreover, the H^1^ NMR spectrum reflected a signal at δ11.1 corresponding to a carboxylic hydrogen.

### Individual extraction of stearic acids

Super critical carbon dioxide extractor was used for individual extraction of pure stearic acid^18,19^. Where, polarity of carbon dioxide in its super critical condition is so sensitive to ward temperature and pressure, thus, it used to be an ideal solvent for fatty acid mixture separation. Thus, stearic acid was extracted individually from the fatty acids mixture at 40.0 MPa and 328 K.

### Synthesis of a new hydrophobic wax (stearyl stearate)

A wax ester is a very long hydrocarbon chain formed through esterification reaction between a fatty acid and a fatty alcohol. The commercially important waxes (Carnauba wax, Candelilla wax, and Beeswax) are mainly made up of wax ester. Wax esters are formed due to so many different possible combinations of fatty acids and fatty alcohols, so, it could be saturated, unsaturated, branched or unbranched hydrocarbon with different chain length, and each combination will have its own characteristics^28^.

Stearyl stearate is a waxy ester formed by combining between stearic acid and stearyl alcohol, stearic acid was previously extracted individually in pure form, while stearyl alcohol was prepared by reducing the corresponding acid^21^. The prepared alcohol was confirmed according to its elemental analysis and spectral data, where its IR spectrum shows disappearance of carbonyl group that is related to carboxylic function at ν 1773 cm^−1^.

**Figure 1:**
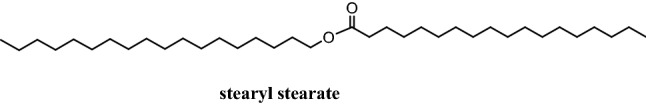
Chemical structure of stearyl stearate.

Then, stearyl stearate (Fig. 1) was prepared by direct esterification between stearic acid and stearyl alcohol in presence of alkaline catalyst, reaction mixture was then washed with warm water in order to get rid of the water-soluble alkaline catalyst, then stearyl stearate was separated from the unreacted fatty alcohol and acid according to its melting point. The separated ester was confirmed according to its elemental analysis and spectral data, where its IR spectrum revealed disappearance of hydroxyl group and appearance of the carbonyl ester at ν 1737 cm^−1^.

### Quality attributes

#### Weight loss

**Figure 2:**
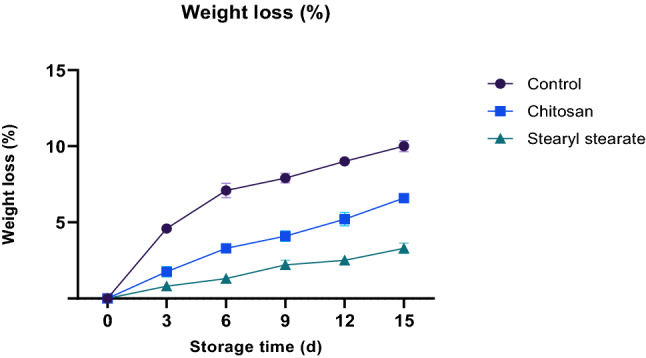
Weight loss of stored tomatoes.

Weight loss caused by water loss from transpiration, respiration, or any other physiological mechanism is considerably delayed by the coating process^29^. When a hydrophobic coating film is used, it serves as a permeable barrier against the flow of moisture and other solutes^30^. Therefore, over the 15-days storage period at 23 ± 1 °C and 65 ± 2% relative humidity, tomatoes coated with hydrophobic stearyl stearate film (2.00% w/v diethyl ether) lost less weight than those coated with chitosan at the same concentration and the non-coated tomatoes (Fig. 2). As a result, it was discovered that for the control sample, tomatoes coated in chitosan, 6.6%, and 3.3% in stearyl stearate, respectively, the percentage of weight reduction was 10.8%, 6.6%, and 3.3%.

#### Changes in pH

**Figure 3:**
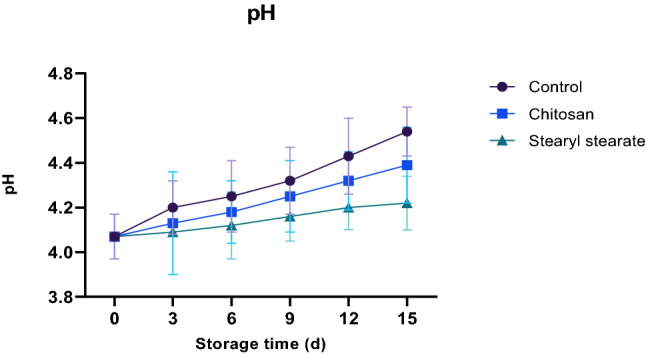
Changes in pH of stored tomatoes.

The presence of organic acids and phenolic compounds, which are recognised for their antioxidant effects, is linked to the acidity of tomatoes. The most common acids in tomatoes are citric and malic acids, whereas lactic, trans-aconitic, fumaric, succinic, citramalic, and pyroglutamic acids are only found in trace amounts. Each acid content may be regarded as a chemical variable for fresh tomato marketing and biological safety^31^. The rate of respiration and metabolic activity in tomatoes are related to pH change^30^ (Fig. 3). Whereas during the process of respiration or conversion into sugars, organic acids are depleted. Coating with stearyl stearate film (2.00% w/v diethyl ether) was found to delay the acidity change more than that caused by both chitosan coating with the same concentration and non-coated tomatoes throughout the storage period of 15 days at 23 ± 1 °C and 65 ± 2% relative humidity as shown in Fig. 3, where this hydrophobic film retards gases permeability, and consequently retards the respiration process that depletes the organic acids. Thus, pH was found to be 4.54, 4.39 and 4.22 for the control sample, chitosan-coated tomatoes and stearyl stearate coated tomatoes respectively.

#### Firmness

**Figure 4:**
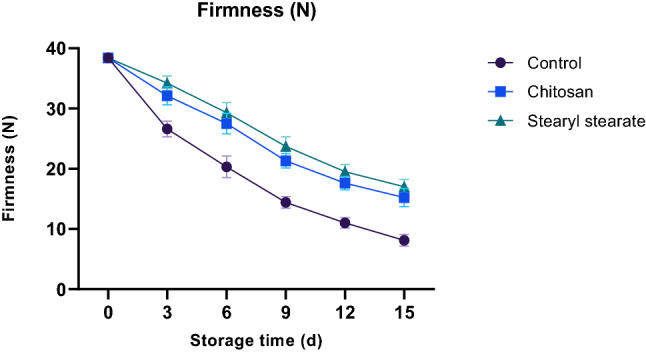
Firmness of stored tomatoes.

Loss of firmness in tomatoes during storage is linked to water loss, microbial development, and cell wall disintegration by endogenous enzymes. While decreased pectin esterase and polylactonase activity, which is the primary cause of the depolymerization of pectin involved in maintaining the cell wall structure, is linked to a loss of firmness in coated tomatoes. This decrease in enzyme activity is brought on by coating films that slow down respiration^32^. It was found that the loss of firmness induced by coating tomatoes with stearyl stearate film (2.00% w/v diethyl ether) was less than that caused by coating tomatoes with chitosan at the same concentration over the 15-days storage period at 23 ± 1.0 °C and 65 ± 2% relative humidity (Fig. 4). For the control sample, chitosan-coated tomatoes, and stearyl stearate-coated tomatoes, respectively, the levels of firmness were determined to be 8.1, 15.2, and 17.0 for each. The hydrophobic stearyl stearate coating is therefore thought to postpone the respiration process, which in turn postpones the activities of pectinesterase and polylactonases, improving the tomato’s interior condition and reducing firmness loss.

#### Ascorbic acid content

**Figure 5:**
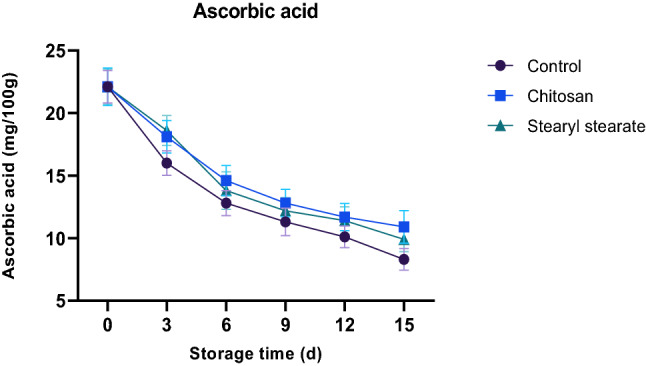
Ascorbic acid content of stored tomatoes.

Ascorbic acid concentration may vary according to the environmental and stress factors such as light intensity, temperature, humidity, air pollution^33^, metabolic conversion of acids into sugars by gluconeogenesis^34^ and its oxidation into l-dehydroascorbat^35^. Thus, coating with the hydrophobic stearyl stearate film (2.00% w/v diethyl ether) retards the respiration process and consequently retards metabolic conversion of acids into sugars and its oxidation into l-dehydroascorbat. Ascorbic acid concentration was found to be 8.3, 10.9 and 9.9 mg/100 g for the control sample, chitosan-coated tomatoes and stearyl stearate coated tomatoes respectively, throughout the storage period of 15 days at 23 ± 1 °C and 65 ± 2% relative humidity, as shown in Fig. 5.

#### Soluble salts percent

**Figure 6:**
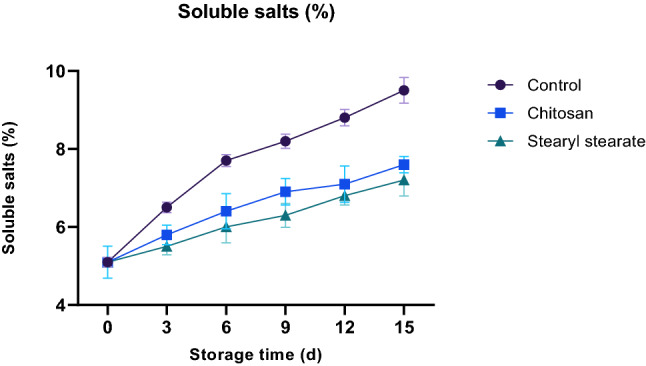
Soluble salts % of stored tomatoes.

During the ripening process, the soluble solids concentrations increased generally due to hydrolysis of polysaccharide to maintain the respiration rate^36^. Whereas in stored tomatoes, slight increases in the TDS level are associated with a decrease in tomato weight resulting from losing some water. Tomatoes coated with stearyl stearate film (2.00% w/v diethyl ether) showed minimum changes in the total soluble solids content compared to that coated with chitosan at the same concentration, and control sample. Total soluble solids were found to be 9.5, 7.6 and 7.2% for the control sample, chitosan-coated tomatoes and stearyl stearate coated tomatoes respectively, throughout the storage period of 15 days at 23 ± 1 °C and 65 ± 2% relative humidity, as shown in Fig. 6.

### Sensory evaluation

For fruits and vegetables, particularly tomatoes, to be consumer-friendly and to be sold, their aesthetic and sensory qualities are crucial. According to Table 3, covering tomatoes with stearyl stearate had a greater positive impact on their appearance, flavour, texture, and general acceptance than did chitosan or a control. As can be observed, the quality of tomato fruit coated with stearyl stearate was the greatest for all quality criteria in comparison to tomatoes coated with chitosan and control samples, respectively, in storage circumstances (23 ± 1 °C/65 ± 2% RH/15 days). Generally speaking, the sensory assessment quality parameters had a significant (p ≤ 0.05) negative impact on the overall acceptability of tomato fruits coated with stearyl stearate.

**Table 3:** Sensory evaluation of stored tomatoes at (23 ± 1 °C/65 ± 2% RH).

Parameters	Stearyl-stearate	Chitosan	Control
Appearance	9.3 ± 0.44^a^	8.8 ± 0.49^ab^	6.6 ± 0.49^c^
Flavor	9.5 ± 0.42^a^	8.60 ± 0.48^b^	5.40 ± 0.49^c^
Texture	9.5 ± 0.44^a^	8.70 ± 0.42^ab^	6.0 ± 0.48^c^
Overall acceptability	9.4 ± 0.43^a^	8.7 ± 0.46^ab^	5.8 ± 0.48^c^

Data are expressed as mean ± SD values given represent means of three determinations; the different letters in the same raw are significant (p ≤ 0.05).

### Acute toxicity test

As the applied dose for applying stearyl stearate in the coating experiment, the toxicity of five graduated concentrations was examined in mice acute test. Table 4 manifested that; by increasing the oral administration dose of stearyl stearate, no mortality was recorded up to 25 g/kg body weight of treated mice. While, a quantity of ~ 50 ml of coated solution was fear enough to apply in the coating process for one kilogram of tomato fruits. This quantity contained ~ 1 g of the stearyl stearate. This amount was still in the green area for the toxicity level due to the obtained results. The daily consumption of tomatoes was reported globally as means of ~ 500 g/day/person^37^, and as means of ~ 350 g/day/person in Egypt^38^. However, the previous data reported a low accumulation rate with the safety and health impact of the stearyl stearate by regular consumption of food materials^39^.

**Table 4:** Acute oral lethal toxicity of stearyl stearate.

Group no.	Dose (g/kg)	No. of animals/group	No. of dead animals
1	5	5	0
2	10	5	0
3	15	5	0
4	20	5	0
5	25	5	0

Experimental animals were divided into 5 groups of mice (5 mice per each).

The applied dose of stearyl stearate was orally administrated up to 25 g/kg body weight. No mortality was recorded for the evaluated groups of mice.

Mice were used for evaluating the cytotoxicity of the new source of fatty materials as a recognized experiment^40^. The result of this experiment referred to the safe dose, which can be applied without the harmful impact of toxicity. Moreover, this experiment could be used for the LD_50_ amount of the applied compound^41^. It was noticed from the result that; up to 25 g of stearyl stearate per gram body weight, no mortality has been recorded in mice. In this regard, it can be concluded that; by application of the stearyl stearate coating for tomato fruits, no restriction could be recorded at the normal daily intake level.

### Liver and kidney function tests

Table 5 shows AST activity of rats feed on different concentrations of stearyl stearate (5, 10, 15, 20, and 25 g/kg). During the whole experiment, AST for rats fed on stearyl stearate were slight non-significant increase. Moreover, similar results were obtained for rat sera activities of ALT and ALP. On the other hand, urea and creatinine contents of rats fed on stearyl stearate at different concentration show very little change on sera levels of urea and creatinine during the whole experiment.

**Table 5:** Liver and kidney function tests of rats feed on tannyl penta stearate at different concentrations.

Parameter groups	ALT (U/l)	AST (U/l)	ALP (U/l)	Urea (mg/dl)	Creatinine (mg/dl)
Control	20.04 ± 0.03	34.99 ± 0.02	137.00 ± 0.02	33.66 ± 0.03	0.754 ± 0.051
5 g/kg	20.46 ± 0.02	35.30 ± 0.02	139.03 ± 0.03	34.66 ± 0.03	0.759 ± 0.003
10 g/kg	20.95 ± 0.05	35.70 ± 0.18	138.04 ± 0.03	34.67 ± 0.02	0.754 ± 0.008
15 g/kg	21.49 ± 0.05	36.10 ± 0.18	140.06 ± 0.05	34.69 ± 0.04	0.763 ± 0.002
20 g/kg	21.81 ± 0.04	36.61 ± 0.02	140.06 ± 0.04	35.31 ± 0.05	0.771 ± 0.002
25 g/kg	22.13 ± 0.03	37.02 ± 0.19	141.10 ± 0.05	35.72 ± 0.02	0.778 ± 0.002

Data are expressed as mean ± SD values given represent means of three determinations.

## Conclusion

The low cost, synthesized, hydrophobic stearyl stearate is characterized with its waxy texture. Moreover, it is formed from a combination of two naturally occurring components. So, it is suitable be considered as a safe food coating compound. And as freshly harvested tomatoes are usually fast spoiled, it was used as a typical example to be coated with a film (2.0% w/v in diethyl ether) of the pervious prepared compound. Thus, the shelf life of tomatoes was extended as this hydrophobic film could control the water permeability through tomato epidermis, thus controlling most of the physiological changes. In addition, this film showed complete safety up to 25 g/kg mice weight. The use of stearyl stearate in coating films is a promising technique that can be used in food applications to increase the shelf life of fast-corruption vegetables and fruits.

### Ethical approval

The experiments were conducted following the ethical guidelines for investigations in laboratory animals and complied with the National Institutes of Health guide for the care and use of laboratory animals (NIH Publications No. 8023, revised 1978). All applicable international, national, and/or institutional guidelines for the care and use of animals were followed.

## Data Availability

The data are available within the manuscript.
